# A multi-parametric analysis of *Trypanosoma cruzi* infection: common pathophysiologic patterns beyond extreme heterogeneity of host responses

**DOI:** 10.1038/s41598-017-08086-8

**Published:** 2017-08-21

**Authors:** Julien Santi-Rocca, Fernando Fernandez-Cortes, Carlos Chillón-Marinas, María-Luisa González-Rubio, David Martin, Núria Gironès, Manuel Fresno

**Affiliations:** 1 Centro de Biología Molecular Severo Ochoa, Consejo Superior de Investigaciones Científicas (CSIC), Universidad Autónoma de Madrid (UAM), Cantoblanco, Madrid, E-28049 Spain; 2 Novancia Business School, F-75015 Paris, France; 3 Instituto Sanitario de Investigación Princesa, Madrid, Spain; 4 Centre de Physiopathologie de Toulouse Purpan (CPTP), INSERM, CNRS, Université de Toulouse, UPS, Toulouse, F-31300 France; 5 Wellcome Trust Centre for Molecular Parasitology and Institute of Infection, Immunity and Inflammation, Present Address: College of Medical, Veterinary and Life Sciences, University of Glasgow, Glasgow, United Kingdom

## Abstract

The extreme genetic diversity of the protozoan *Trypanosoma cruzi* has been proposed to be associated with the clinical outcomes of the disease it provokes: Chagas disease (CD). To address this question, we analysed the similarities and differences in the CD pathophysiogenesis caused by different parasite strains. Using syngeneic mice infected acutely or chronically with 6 distant parasite strains, we integrated simultaneously 66 parameters: parasite tropism (7 parameters), organ and immune responses (local and systemic; 57 parameters), and clinical presentations of CD (2 parameters). While the parasite genetic background consistently impacts most of these parameters, they remain highly variable, as observed in patients, impeding reliable one-dimensional association with phases, strains, and damage. However, multi-dimensional statistics overcame this extreme intra-group variability for each individual parameter and revealed some pathophysiological patterns that accurately allow defining (i) the infection phase, (ii) the infecting parasite strains, and (iii) organ damage type and intensity. Our results demonstrated a greater variability of clinical outcomes and host responses to *T. cruzi* infection than previously thought, while our multi-parametric analysis defined common pathophysiological patterns linked to clinical outcome of CD, conserved among the genetically diverse infecting strains.

## Introduction

In Latin America, 110 million people are at risk of contracting the life-threatening Chagas disease (CD) caused by infection of the protozoan *Trypanosoma cruzi* ^1^. Subsequent to global warming and human migrations, this disease is now a global public health issue^2, 3^. CD presents various clinical outcomes: spontaneous healing, asymptomatic infection, cardiac, visceral, or cardio-visceral forms. Besides the worldwide effort to stop CD spreading, there is a lack of reliable diagnostic, prophylactic, and therapeutic tools.

A major issue for the efficient control of CD is the different clinical outcomes of infection. Indeed, after an acute infection, symptomatic or not, patients can evolve either to spontaneous cure or to an “undetermined”, asymptomatic stage from which they can switch to CD *stricto sensu*, with clinically different cardiac, digestive, or cardio-digestive forms^1^. Mostly based on epidemiological findings, it has been proposed that this heterogeneous clinical response is linked to *T. cruzi* extremely high genetic diversity^4^. The species *T. cruzi* has been divided into six major genetic discrete typing units (DTU): TcI to TcVI, reflecting this heterogeneity^4, 5^. *Trypanosoma cruzi* can infect rodents in nature and murine models mimic the different steps of the CD in humans, even producing different clinical outcomes. Differences observed in organ damage were proposed to be related to the genetic diversity of *T. cruzi*. Initial experiments have shown that tissue tropism depends on parasite strains^6^, where pro-inflammatory responses can be triggered by first line defense, phagocytic cells^7^. Since high levels of pro-inflammatory cytokines in patients are linked to cardiac damage^8^, excessive pro-inflammatory responses are thought to be detrimental for host tissues. However, a direct correlation between levels of parasite load and damage in the tissues is not entirely clear, since there are some description of parasite-triggered autoimmunity that can damage organs even in the absence of great parasitization^9–12^. The balance between these responses was proposed to partially depend on the genetic determinants of the parasite. This was based on epidemiological findings showing association of defined DTUs of the parasite with severe cardiomyopathic manifestations in patients^4, 13–15^ as well as in animal models^16^, although other studies found no associations^17^. Additionally, the infectious background of the host was assessed in co-infection experiments with different strains of *T. cruzi* showing differences in tropism^18^, while former, cured infection can impact response in case of re-infection^19^. Altogether, these data evidence important gaps in the current knowledge of CD pathophysiology and suggest that the outcome of the disease depends on various parameters, among which, the genetics of the parasite should be considered.

To address this issue, we have studied pathophysiological features of CD in a murine infectious model system by using *T. cruzi* strains from genetically diverse DTUs, providing a holistic description of experimental infection. We used a multi-parametric approach to gather and integrate a high number of pathophysiological markers. Here, we show that the infecting strain of the parasite consistently impacts CD outcome. However, variability between individuals – even infected by the same strain – was high and hindered reliable one-dimensional clustering in groups. A multi-dimensional statistical analysis overcame this issue and showed that combinations of parameters accurately predicts infection phase, infecting strain, and clinical presentation of the disease. This revealed an extreme heterogeneity of host responses, both in the acute and chronic phases. However, while a combination of factors allow the identification of the infecting strain, some patterns are conserved during CD pathophysiogenesis across the whole *T. cruzi* species, despite its genetic diversity.

## Results

To reflect and maximize the diversity of the parasite genetic background, we infected syngeneic mice with 6 clonal *T. cruzi* strains (X10 (Sylvio X10), Esmeraldo, CM17, 10R26, Sc43, and VFRA), one for each DTU (I to VI, respectively), and compared parasitological pathophysiological and immunological parameters between mock controls, acute, and chronic phases. All individual results and corresponding statistics are presented in tables, focusing on comparison between infected and control animals (Table S1), phases of infection (Table S2), parasite strains (Table S3), parasite strains for each phase of infection (Table S4), intestinal damage intensity groups (Table S5), and cardiac damage intensity groups (Table S6).

### Organ damage

We found detectable parasitemia with VFRA strain, with a peak at 28 days post-infection (dpi) (Figure S1). Thus, we used this time point as the reference for acute phase for all the strains that coincides with an accepted time for the acute phase^12^. No circulating parasites were detected with VFRA strain from 60 dpi. In agreement with previous studies, we set 84 dpi as the time point for chronic phase^20–22^.

**Figure 1:**
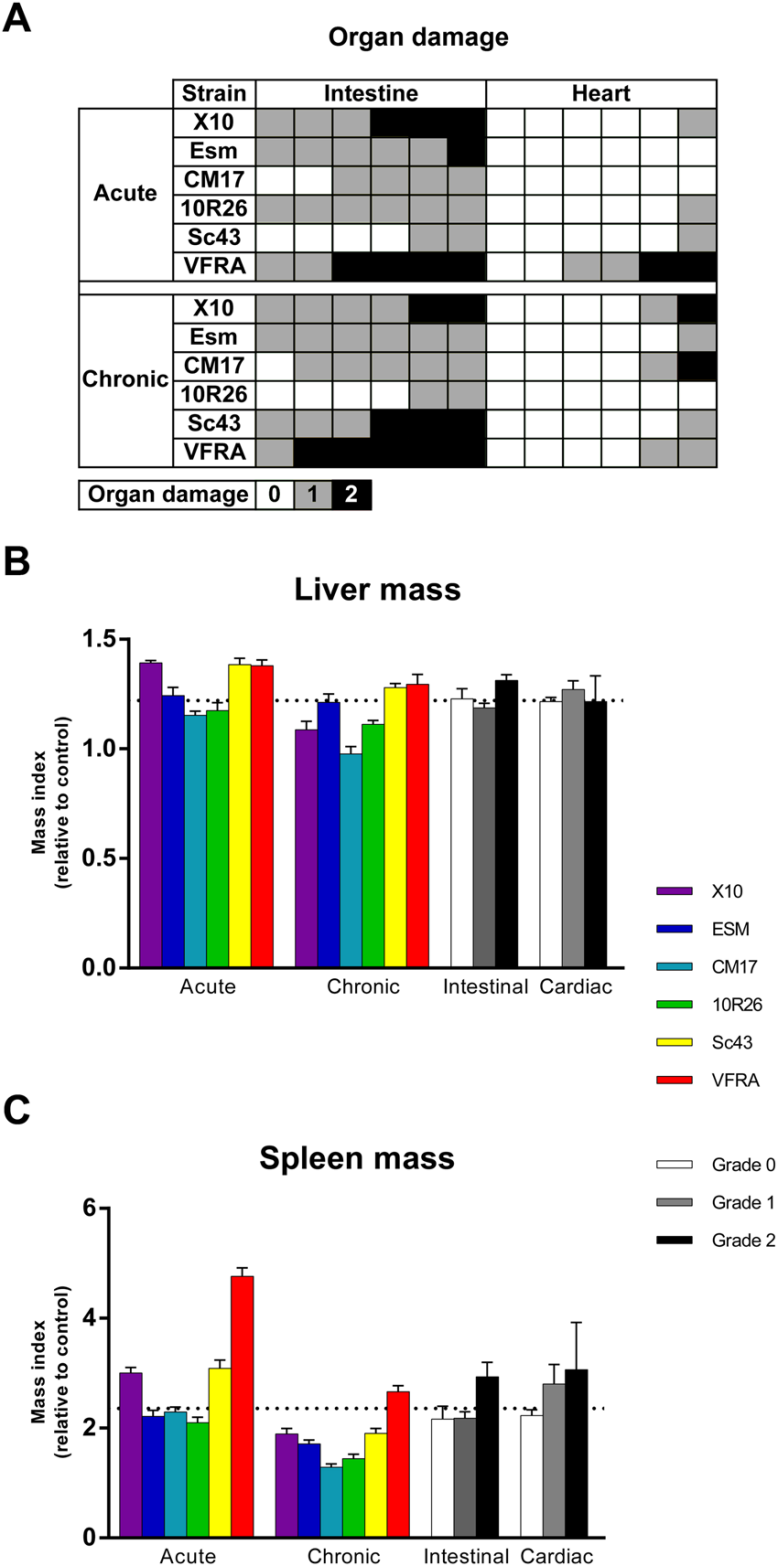
*Trypanosoma cruzi* strains differently impact organs during acute and chronic infection. (**A**) Damage to intestine and heart vary among strains and phases. Damage was assessed upon dissection by macroscopic observation and resistance to pressure and extension, resulting in a 3-grade scale: 0, unaffected (white), 1, low damage (gray), 2, high damage (black). Each animal is represented by a coloured square. Hepatosplenomegaly is a common feature of both acute and chronic phases, with sensible differences among strains. (**B**,**C**) Mass index is the weight of infected animals normalized to controls, grouped per strain and per phase (coloured bars), and according to organ damage (white, gray, and black bars) for liver (**B**) and spleen (**C**). Dotted lines stand for the mean of all infected animals. Data are the mean per group ± S.E.M.

To study the clinical outcome in this CD model, organ damage was sought during dissection, at the indicated time points, for key organs: intestine and heart. Macroscopic changes were observed as white stripes on the cardiac muscle and remarkable changes in texture and appearance. Similarly, intestinal macroscopic damage was observed at dissection as a loss of elasticity of the colon leading to rupture upon extension, as well as changes in the volume and tissue color. We categorized these observations into three levels of organ damage: no, low, or high (indexes 0, 1, and 2, respectively). Results of macroscopic observations are shown in Fig. 1A. Moreover, histological observations were correlated with macroscopic discrimination, allowing a damage classification for both intestinal and cardiac damage (Figure S2).

All strains significantly produced intestinal damage, as compared to mock controls, but did not follow a common pattern for all the strains **(**Table S3). Interestingly, when analyzing in more details by phase, Sc43 in acute phase and 10R26 in chronic phases did not produce consistent damage as compared to controls (Table S4). Thus, infection by 10R26 parasites triggered intestinal damages during the acute phase that cure (p = 2.46 × 10^−2^; Table S4) in sharp contrast to Sc43 in which damage appeared later during the chronic phase (p = 1.30 × 10^−2^).

Cardiac hypertrophy was not detected (data not shown) and heart damage was weaker than in the intestine for all strains. Interestingly, some consistent differences were observed between the strains (Table S3). Indeed, while only 1 animal out of 12 infected with 10R26 or Sc43 developed cardiac damage, half the animals infected with VFRA were affected (p = 2.73 × 10^−2^ in both cases; Table S3).

Thus, this experimental strategy was compliant with our attempt to obtain diversity in CD outcome using supposedly distant *T. cruzi* strains from genetically different DTUs.

Hepatosplenomegaly is a common feature of *T. cruzi* infections in human, either in acute or chronic phases. Splenomegaly was observed with all strains in both phases (Fig. 1C), however with noticeable differences between phases and strains (Tables S2 to 4). Hepatomegaly (Fig. 1B) was also common, except for CM17 strain in chronic phase (p = 5.44 × 10^−1^; Table S4). Interestingly, although hepatomegaly was generally more pronounced in acute phase (p = 7.45 × 10^−5^; Table S2), its degree was not significantly modified between acute and chronic phases for half the strains: Esm, 10R26, and VFRA (p = 5.60 × 10^−1^, p = 1.55 × 10^−1^, p = 1.38 × 10^−1^, respectively; Table S4).

Hepatosplenomegaly was particularly strong in animals with high intestinal damage as compared to those with low damage (p = 1.18 × 10^−3^ and p = 4.37 × 10^−3^, for hepato- and splenomegaly, respectively; Table S5). Surprisingly, hepatosplenomegaly was not significantly higher in the high heart damage group as compared to controls (p = 1.68 × 10^−1^ and p = 9.59 × 10^−2^, for hepato- and splenomegaly, respectively; Table S6), mainly due to high variability in this group, in particular for splenomegaly (coefficient of variation = 0.486).

These results revealed that hepatosplenomegaly was linked to infection, but did not allow predicting accurately the extent of organ damage.

### Parasite tropism

**Figure 2:**
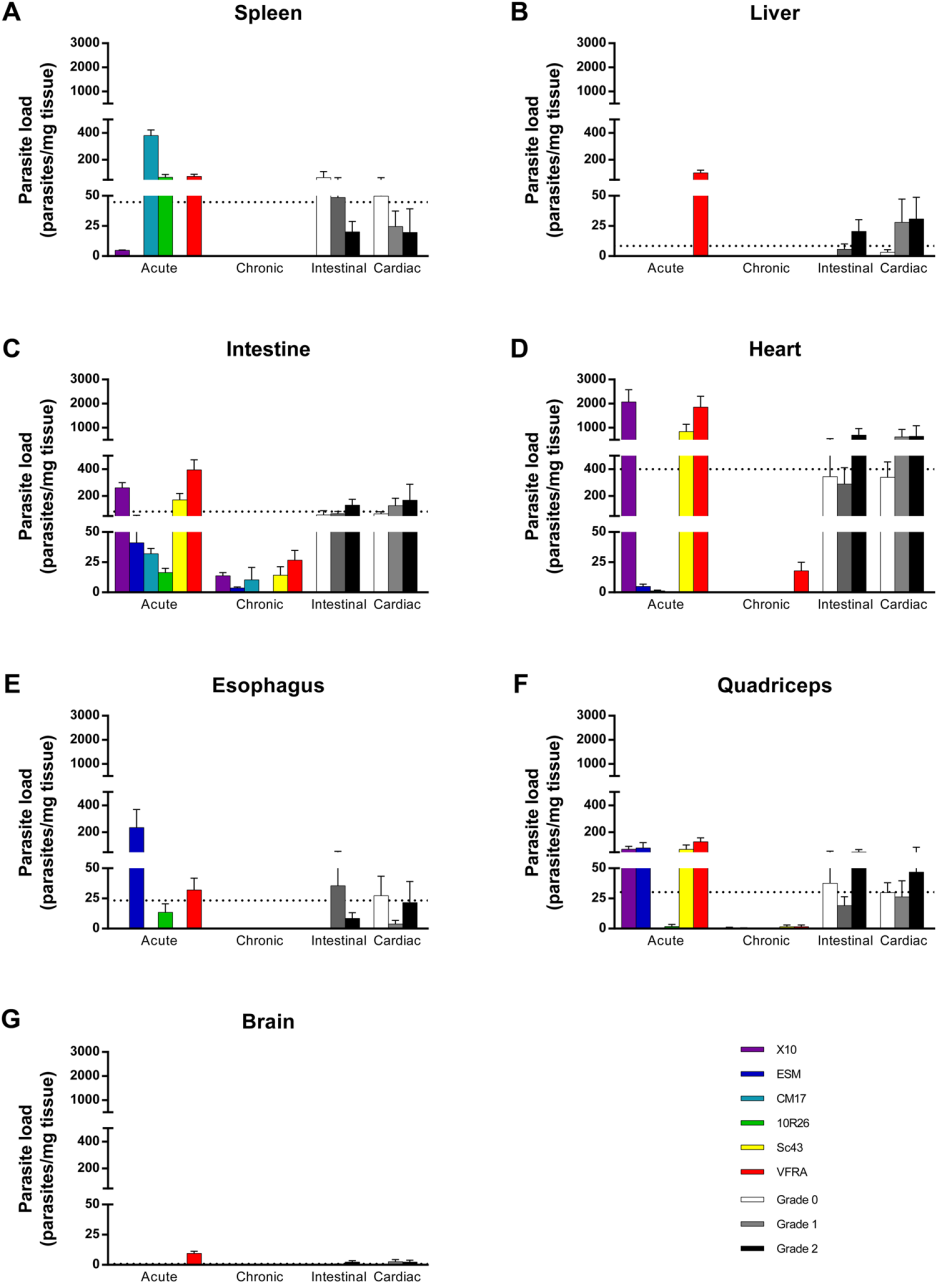
*Trypanosoma cruzi* strains exhibit different organ tropism during acute and chronic infection. Parasite load was measured by qPCR and expressed as the number of parasites per mg of tissue. Are presented animals grouped per strain and per phase (coloured bars) and according to organ damage (white, gray, and black bars). Data are the mean of the groups ± S.E.M. Dotted lines stand for the mean of all infected animals. Individual data are presented in Tables S1 to S6.

To gain further insight into differences or similarities between strains, and their impact on the pathophysiology of the disease, we measured by quantitative PCR the parasite burden in organs (Fig. 2). In acute phase, tropism, measured as parasite load, exhibited high divergence between parasite strains (Table S4). Common trends could be drawn: presence of parasites in the spleen, intestine, heart, and quadriceps; their absence in brain and liver. However, some exceptions were noteworthy: during acute phase, parasites were not detected in the spleen for Esm and Sc43, in the heart for 10R26 and in skeletal muscles for CM17, while only VFRA parasites were detected in brain and liver during acute phase.

Despite this high divergence in acute phase, chronic phase was more homogeneous for all strains and characterized by an absence of parasites in the spleen, liver, esophagus, and brain for all the strains. In the intestine, only 10R26 parasites were totally cleared, as well as in skeletal muscles (quadriceps), as for CM17 strain. In the heart, only VFRA parasites were not totally cleared in chronic phase: all animals had a cardiac parasite burden, but with a difference of two orders of magnitude as compared to acute phase (p = 9.19 × 10^−3^; Table S6). Interestingly, no differences in parasite load were observed relative to organ damage, except for liver in animals with high intestinal damage, as compared to infected animals with no damage (p = 4.72 × 10^−2^; Table S5).

A striking dichotomy was observed between acute and chronic phases: while cardiac damage was positively correlated to heart parasitism in acute phase (6 animals with detectable cardiac parasite load out of 7 animals with damage), it was not the case in chronic phase (2 out of 8).

Intestinal damage was characterized by higher intestinal load (as compared to control; p = 1.63 × 10^−5^; Table S5). It is noteworthy that differences in intestinal parasite load between acute and chronic phase (means for each phase are 157 and 11 parasites/mg tissue, respectively; p = 1.23 × 10^−5^; Table S2) contribute to the high variability in intestinal damage classes (coefficients of variation are 1.68, 1.72, and 1.38 for classes 0, 1, and 2, respectively; Table S5). By treating phases separately, we could highlight that higher acute intestinal damage was linked to higher parasite load (means are 125 and 277 parasites/mg tissue for classes 1 and 2, respectively, p = 3.41 × 10^−2^). In contrast, damage in chronic phase was correlated to the presence of parasites (24 animals out of 31, p = 1.61 × 10^−4^), but damage grade is not linked to the extent of parasite load (means are 12.7 and 14.4 parasites/mg tissue for classes 1 and 2, respectively, p = 7.54 × 10^−1^).

Altogether, the above results underlined that parasite tropism was influenced by strain-linked determinants, by the infection phase, and that tropism was in some cases linked to organ damage. However, a strict classification in these categories using discriminant thresholds for parasite load in organs was impossible (data not shown), highlighting the intricate pathophysiology of CD and the need for further studies to understand it.

### Immune responses

**Figure 3:**
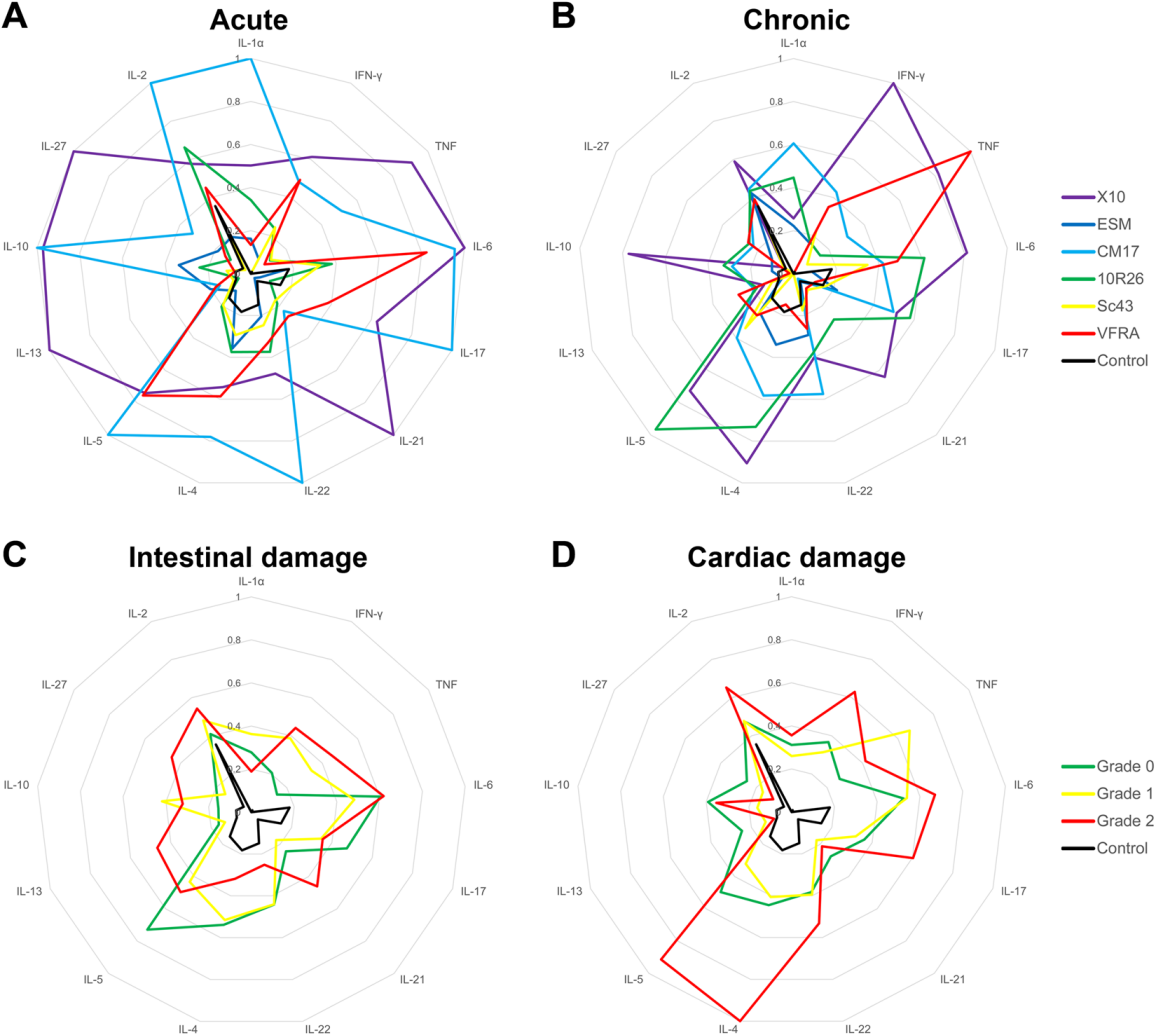
Serum cytokine concentrations and patterns differ between infection phases, and *T. cruzi* infecting strains. Cytokine concentration was measured by multiplex bead array assay in sera from acute (**A**) and chronic (**B**) phases, and expressed as a ratio to the highest group mean. Results are also grouped by intestinal (**C**) and cardiac (**D**) damage extent. Data are presented as the mean of each group. Absolute concentrations in pg/ml are presented in Tables S1 to S6.

Cytokine concentration measurement in the sera of infected mice revealed that a strong systemic immune response was induced by all parasite strains. Interestingly, all cytokines, except IL-13, were increased in the sera of infected mice in comparison to control animals, (Table S1). Average values *per* strain and phase allowed us to determine different profiles, highlighting striking differences (Fig. 3). Some expected correlations between cytokines were not always conserved: for instance, TNF and IFNγ serum levels were weakly correlated in acute phase (R^2^ = 0.276) and not at all in chronic phase (R^2^ = 0.097). This correlation was particularly affected by the parasite strains.

It is noteworthy that some cytokines were associated to high damage, as higher IL-4 concentration to high cardiac damage (p = 3.89 × 10^−2^, Table S6 and Fig. 3D), whereas lower IL-22 concentration was associated to high intestinal damage (p = 9.46 × 10^−3^, Table S5 and Fig. 3C). Though these associations are consistent, no single serum cytokine allowed to set a threshold to distinguish accurately acute from chronic phases, or strains between them. Similar observation was already made with sera from Chagasic patients^23^.

**Figure 4:**
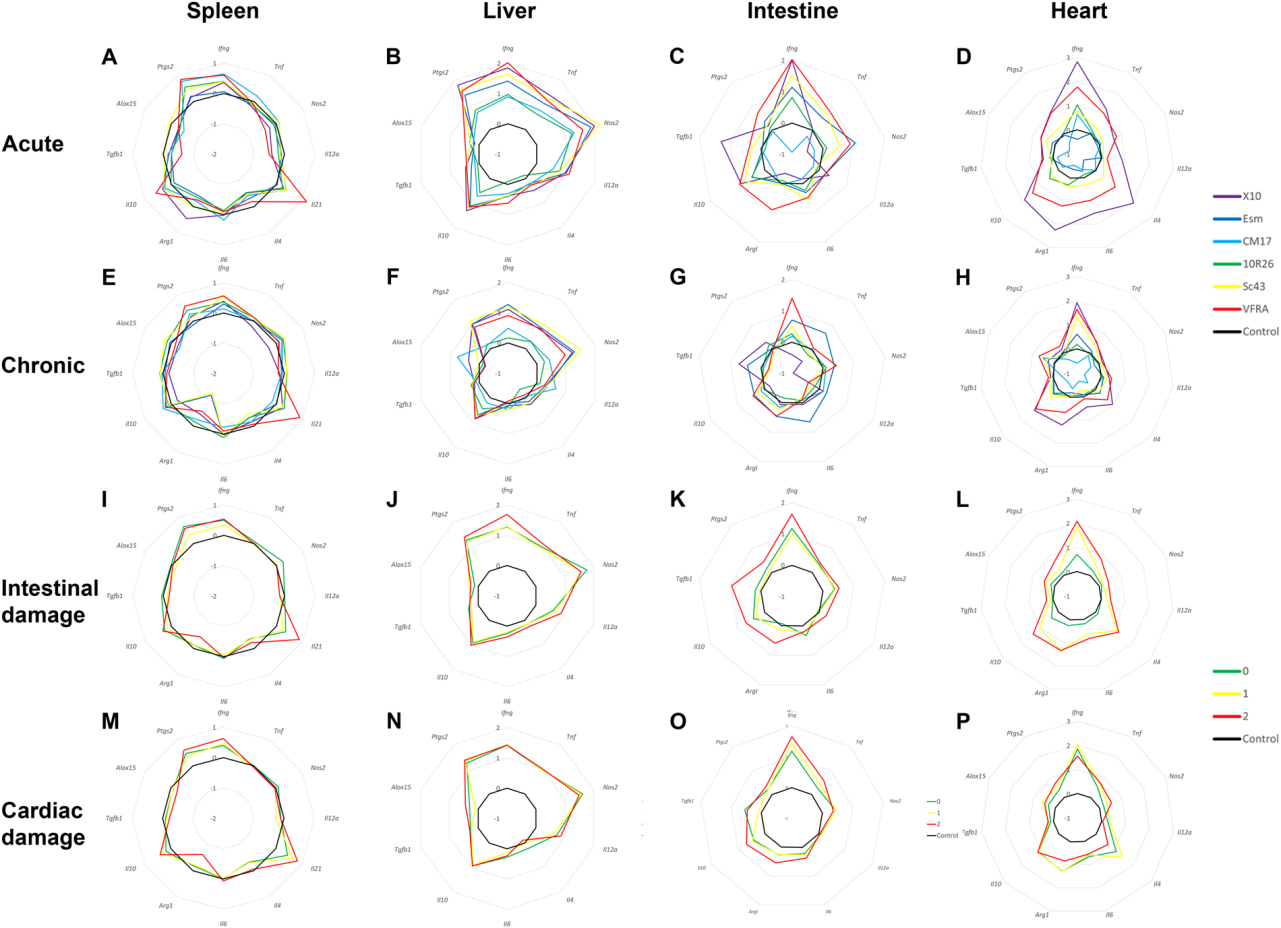
Gene expression levels and patterns differ between infection phases, organs, and *T. cruzi* infecting strains. Gene expression was measured by qRT-PCR in the spleen (**A**,**E**,**I**,**M**), liver (**B**,**F**,**J**,**N**), intestine (**C**,**G**,**K**,**O**), and heart (**D**,**H**,**L**,**P**). Animals are grouped by strains in acute (**A–** **D**) and chronic phases(**E**–**H**), and by intestinal (**I**–**L**) or cardiac (**M**–**P**) damage. Gene expression was normalized to uninfected controls (black lines). Data are presented as the log_10_ of the group mean (see Tables S1 to S6 for details).

To analyze local responses, we studied the cytokine gene expression in two organs known to be involved in the immune response to *T. cruzi* infections, spleen and liver^19^, and in two organs undergoing damage, heart and intestine (Fig. 4 **)**. Strikingly, most genes were modulated in infected animals – irrespective of the parasite strain, phase, or damage – as compared to uninfected controls (Table S1 and Figure S3). Responses were diverging in an extreme way between organs and no rule could be drawn to predict response in an organ from the expression pattern in another.

The acute splenic response (Fig. 4A) was characterized by an upregulation in infected animals of *Ifng*, *Il10*, *Il21*, and *Ptgs2* with a downregulation of *Il4*, *Alox15*, *Tgfb1*, and *IL12a*, as compared to controls (Table S2). Beside these common markers, some genes were modulated in animals acutely infected by specific strains: *Tnf* was downregulated by X10 infection, *Nos2* for X10 and VFRA, *Arg1* for Esm and 10R, while infection with CM17 and Sc43 strains did not lead to modulation of *Tgfb1* gene expression (Table S4).

In chronic phase (Fig. 4E), *Nos2* was upregulated in the pool of infected animals. In contrast to acute phase, *Il12a*, *Tgfb1*, and *Alox15* genes were not modulated in chronic phase, while *Arg1* was downregulated (Table S2). This gene expression analysis performed on whole organs could not identify a splenic immune response polarization in response to acute or chronic infection. However, downregulation of splenic *Arg1* expression was a common feature in animals with high damage to either the intestine or the heart as compared to controls (p = 6.11 × 10^−3^ and p = 2.38 × 10^−3^, Tables S5 and S6, respectively). This enzyme is synthesized by myeloid-derived suppressor cells and involved in T cell suppression^20, 24^, thus highlighting possible bottlenecks for the orientation towards a given clinical outcome.

We also undertook the analysis of hepatic gene expression in response to *T. cruzi* infection (Fig. 4B and F). Responses were similar among strains, almost homothetic, and all genes were upregulated in infected animals as compared to controls. However, there were differences in the magnitude of the response to different strains, which were not correlated to the extent of parasite load in this organ, suggesting a role for immune modulation from other tissues.

In the intestine of infected animals, the expression of *Ptgs2*, *Ifng*, *Il10*, *Il6*, *Nos2*, and *Tnf* was upregulated in both phases (Fig. 4C and G, Table S1). Additionally, *Arg1* gene expression was upregulated in chronic phase (p = 1.54 × 10^−2^, Table S2). It is noteworthy that *Tnf* gene expression in chronic phase was differentially modulated according to the infecting strain: downregulated for X10 and 10R26, while upregulated for Esm (p = 5.93 × 10^−5^, p = 2.49 × 10^−2^, p = 9.90 × 10^−3^, respectively, Table S4) and invariant for other strains. Strikingly, *Ifng*, *Il10* and *Tnf* gene expression was consistently upregulated in animals with mild and severe intestinal damage (p = 1.95 × 10^−4^, p = 2.26 × 10^−5^, p = 6.16 × 10^−4^, Table S5). These observations suggest a role of these biomarkers during the pathogenesis of intestinal CD, which should be checked with appropriate experiments.

Upon infection, expression of all analyzed genes was induced in the heart (Table S1) and patterns were conserved between acute and chronic phases (Fig. 4D and F, respectively), with only few exceptions. Indeed, cardiac *Arg1* expression was unexpectedly downregulated in some acutely infected animals, in particular in response to CM17 infection (2.13 × 10^−3^, Table S4), while it was strongly upregulated upon X10 and VFRA infection, as previously described with the Y strain^20, 24^. However, this upregulation was not statistically significant using Student’s T test, due to high intragroup variability possibly linked to non-linear effects. Furthermore, gene expression profile of animals infected by VFRA strain was almost opposite to these provoked by CM17 parasites, both strains being cardiopathogenic. Interestingly, cardiac *Alox15* expression did not differ in animals chronically infected with both these cardiopathogenic strains (p = 4.37 × 10^−1^, Table S4), and was linked to cardiac damage (p = 1.84 × 10^−2^, Table S6 and Fig. 4P).

This example of the extreme heterogeneity of *T. cruzi* infection may explain the different types of cardiac alteration observed among strains in mice at the molecular or immunological level^16^, which is also a reflection of what is observed in chagasic patients^23^. A deeper analysis revealed that intestinal upregulation of *Arg1* expression is observed in animals with intestinal damage (p = 3.00 × 10^−3^, Fig. 4K and Table S5), while this gene’s expression is downregulated in the spleen of animals with high cardiac damage (p = 4.76 × 10^−2^, Fig. 4M and Table S6). These observations underline the importance of this gene for the pathophysiology of CD.

By this broad analysis, we have shown that some common markers of the pathology were conserved independently of the infecting strains. In addition, we found that parasitological and pathophysiological parameters, as well as immune responses are extremely variable among *T. cruzi* strains, more than previously suspected, as well as between phases and organs. While some factors consistently vary between phases, strains, and organ damage, their predictive power is low using uni/bivariate statistics. Indeed, correlations between two parameters (Pearson’s correlation coefficient) or comparison between groups (Student’s T test or Mann-Whitney-Wilcoxon’s U test), failed to highlight discriminating parameters or to induce the construction of simple classification algorithms or trees.

### Multi-parametric associations with infection phases, strains, and organ damage

Variability in responses to *T. cruzi* infection has until now impeded precise diagnosis based on biomarkers of infection and renders difficult a general description of CD pathophysiogenesis. We addressed this issue by using the dataset resulting from our wide study, focusing on determining rules for classification in biologically or medically relevant groups: phases of infection, parasite strains, organ damage and grade. We employed the dedicated linear discriminant analysis (DA) to identify combinations of the 64 measured parameters to form groups (the intestinal and cardiac damage parameters were not used for the DA), like in the founding example of Fisher^25^ and in a previous study from our team^23^.

**Figure 5:**
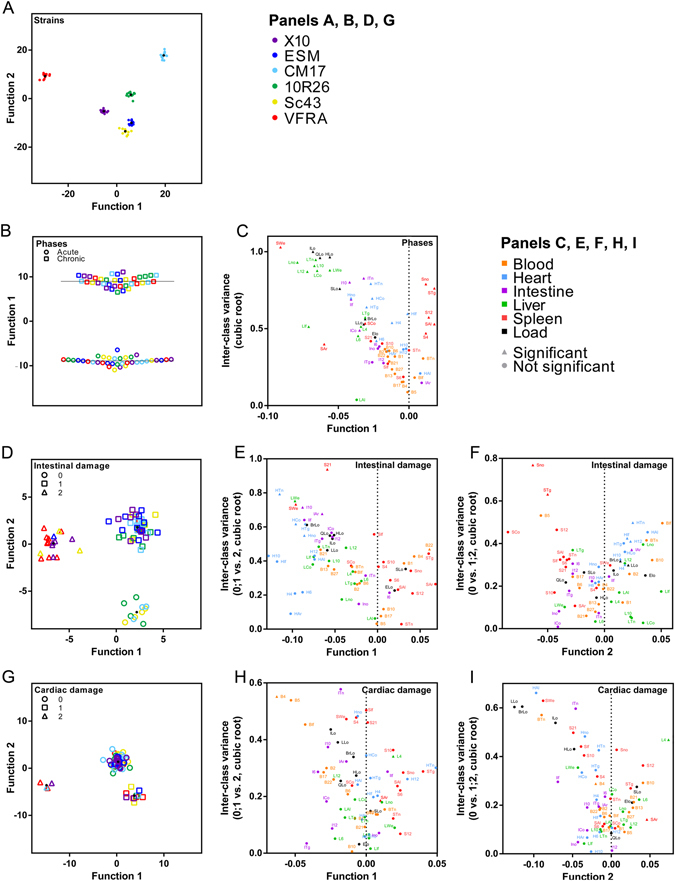
Infection parameters present common determinants evidenced by linear discriminant analysis. Infecting parasite strains (**A**), phase (**B**) intestinal (**D**) and cardiac (**G**) damage can be efficiently grouped using one-dimensional (**B**) or two-dimensional (**A**,**D**,**G**) plotting. Each element stands for an animal; points stand for centroids of the group in 2-dimensional graphs; dotted lines stand for the mean of the groups in panel B. Functions used for plotting were calculated by discriminant analysis; their matrices of structure are presented in Table S7. Panels **C** (for panel **B**), **E** and **F** (for panel **D**), **H** and **I** (for panel **G**) present the correlation of the parameters to the discriminant function (x axis) and the corresponding inter-class variance of the indicated groups, presented as its cubic root for visualization purposes. Triangles indicate variables whose mean was significantly different between the two indicated groups; points stand for no differences in their means.

In our study, linear DA allowed to place each animal strictly in the right group (Fig. 5), as observed in 1 dimension for phases (Fig. 5B), 2 dimensions for strains, intestinal and cardiac damages (Fig. 5A,D and G, respectively). A detailed analysis of these functions is presented in Text S1.

Acute phase correlated to hepatomegaly and splenomegaly- as expected - but also to liver response and to parasite load in organs (negative values in the structure matrix, and Fig. 5C). Indeed, these parameters were consistently upregulated in this phase (Table S2). Interestingly, splenic response was evidenced as protective against high intestinal damage (Function 1, positive values, Fig. 5E), as opposed to cardiac response. On another axis, hepatic, cardiac, and blood responses – in particular pro-inflammatory ones – correlated with damage (Function 2, positive values, Fig. 5F). Inversely, splenic and, to a lower extent and in an inhomogeneous fashion, intestinal responses were linked to absence of damage (Function 2, negative values, Fig. 5F).

Remarkably, strong cardiac damage correlated with blood and intestinal responses (Function 1, negative values, Fig. 5H). As for the intestine, splenic response was inversely correlated to high damage (Function 1, positive values, Fig. 5H). Cardiac damage was finally linked to parasite load in various organs, hepatosplenomegaly, and intestinal expression of pro-inflammatory genes (Function 2, negative values, Fig. 5I). Interestingly, heart response was unevenly correlated with damage.

This analysis confirms that common pathophysiogenesis patterns can be detected from animals developing comparable damage, despite being infected with genetically heterogeneous strains. Combinations of factors, in particular cytokines in serum, may be useful for diagnosis in humans as previously discussed^23^. More importantly, these combinations allowed, in the present murine model, the accurate grouping of individuals (100% accuracy for each presented function combination) and revealed the importance of factors that failed previous univariate (using only one parameter for mean or rank comparison) tests. This underlines the importance of the contribution of each parameter, witnessing the fine-tune regulation of pathophysiological events leading to CD outcome.

## Discussion

Since the first description of American trypanosomiasis by Dr. Carlos Chagas, increasing evidence indicate the complexity of this human disease, although the reasons for this are not entirely clear yet^1, 2^. Moreover, few studies in animal models have scarcely addressed this heterogeneity and few studies have been performed with more than 2 different strains^6, 12, 14, 18, 26, 27^. To address the heterogeneity of CD, we infected animals with 6 *T. cruzi* strains – one from each DTU of the species – and analyzed 66 markers related to the pathophysiogenesis, thus covering a wide range of host responses in a multi-parametric approach. To better characterize CD pathophysiology, we did not focus only on comparison of central tendencies between strain groups, but looked for common patterns using multivariate statistics (discriminant analysis) and individual mice. Our analysis revealed extreme heterogeneity of host responses, strain-specific pathophysiological signatures, and organ damage-linked biomarker patterns conserved across the *T. cruzi* species. The statistical approach we used was first designed by Fisher’s pioneering contribution to classify plant species^25^. In this study, we followed this seminal path and confirmed by a cross-disciplinary approach that it could be beneficial for a variety of systems.

Our study provided novel and original information on the pathophysiogenesis of CD. Indeed, we observed that heart damage is not always linked to parasite load; additionally, parasites can be present without damage. On the contrary, intestinal damage is associated to parasite load. Thus, intestinal damage is linked to parasite persistence while cardiac damage is not (in particular in chronic phase), indicating that – at least in mice – intestine is the preferential site for parasite persistence in the chronic phase, in accordance with recent reports^12^. This underlines the need to better understand the pathophysiology of *T. cruzi* infections, and to identify the possibilities for switching from parasite-induced damage to other mechanism of damage, as parasite-triggered autoimmunity/inflammation/deleterious immune response^9^. To determine the causative relationships in infection events, which are still a black box, future modelizations will need intensive cross-conditional experimentations. Determining the place (organ), the time (course of infection), the actors (cell types), and the communication code used (*i.e*. gene and cytokine expression) will allow to decipher the bittersweet dialogue that leads to disease (damage).

Biomarkers in CD to monitor disease progression and response to therapy are desperately needed^28–30^. Therefore, we searched for those potential biomarkers. Regarding circulating cytokines, IL-4 was consistently higher in the blood of animals with high cardiac damage, and IL-22 was in significant lower concentrations in mice with high intestinal damage. In addition, our study identified 2 genes whose expression was modulated in organs with damage: *Alox15* in the heart and *Arg1* in the intestine. However, our broad analysis revealed a greater heterogeneity than expected and previously described for all measured parameters, impeding threshold-based uni-parametric diagnosis strategies. This can be due to our experimental setup, in which we used low, close to natural infection doses (2,000 parasites/mice) and to the use of parasites not adapted to animal infection by recurrent passages. Thus, some parameters are consistently modulated in damage groups but cannot be used alone for diagnosis due to their high variability among individuals. Nonetheless, these parameters of interest, though not powerful for a threshold-based discrimination, may be used in multi-parametric strategies as markers for cure in preclinical animal models, in particular for treatment discovery process^31^. Moreover, coinfection by several strains is becoming a common feature in human CD^17, 32, 33^.

The presence of tightly modulated gene expression indices with extreme outliers is not surprising in networks resulting from the integration of innumerable stimuli coming from different tissues and cell types. Nonlinear interactions in gene expression networks can thus occur (plateau, threshold, Boolean responses, multi-level integration of stimuli…), accounting for non-normal distribution of values coming from biological replicates. Thus, classical diagnosis using a single *bona fide* candidate and an empirically determined threshold to discriminate categories seemed to be unsuitable for CD.

In a previous study, multivariate methods allowed us to discriminate between cardiac and non-cardiac chagasic human patients^23^. In agreement with that, using discriminant analysis of principal components, we have previously shown that a small set of biomarkers (serum cytokines) is sufficient to have an acceptable diagnostic power, bypassing heterogeneity of responses due to the multi-parametric nature of the stimuli that cause them. Simulations with our murine dataset indicate that comparable results could be obtained using only serum cytokine data (data not shown). However, to gain more precision both in description of the pathophysiogenesis and diagnosis, we used a holistic approach in which all parameters were submitted to multivariate statistical analysis. Linear discriminant analysis of the 64 parameters was performed in different conditions, for discrimination of infection phase, parasite strain, and clinical outcome (including grades of damage of various organs). This strategy led to the identification of common patterns linked to clinical presentation of the disease regardless of the parasite strain, which was initially unexpected, since clinical presentations and pathophysiological parameters were extremely variable among animals infected even with the same strain of *T. cruzi*. Thus, multivariate statistics identified common patterns associated to damage, strain, and infection phase, that seem to be more efficient for use as biomarkers, in particular for diagnosis. In agreement with this, new studies in human CD patients indicate that Tc types and clinical manifestations of disease are not associated^17^. In summary, our studies support the use of combined parameters as a way to circumvent parasite- and phase-dependent heterogeneity in CD diagnosis, identifying common patterns suitable for monitoring infection. Paradoxically, our analysis highlights the extreme heterogeneity of host responses to the infection by the parasite, but also provides for the first time a set of common traits that may unify the species *T. cruzi* beyond their diversity of responses and highly diverse genetic background.

Trees do not allow seeing the wood: heterogeneity of responses to *T. cruzi* at immunological and pathophysiological levels was described both in murine models^16, 26, 27, 34^ and patients^23^ but not clearly integrated into a model compliant with clinical situations. For instance, the same treatment is applied to all infected humans regardless of the clinical manifestations or the infecting strains, despite knowing that all strains may not be equally susceptible to drugs^35, 36^. In clinical conditions, direct parasite genotyping is difficult or not feasible in patients with chronic CD, since parasites are rarely detected in blood or few in organs (as evidenced in this study in animals). Our approach made possible accurate identification of the infecting strain without targeting any parasite markers in the animal; its transfer to humans may help in finding new diagnosis strategies and concepts for health professionals in CD, eventually applicable for personalized therapeutic management. To this goal, further studies taking into consideration other parameters impacting CD development will be essential, like these linked to the host (for instance: age, sex, genetic and immunological backgrounds) and to the infection itself (for instance: dose, site, vectorial or inter-human).

In conclusion, we document here the extreme heterogeneity of host responses to *T. cruzi* in an animal model: this heterogeneity was observed between parasite strains and between animals infected with the same parasite strain. However, multi-parametric analyses allowed defining pathophysiological signatures specific to strains and organ damage across the *T. cruzi* species. Our results highlighted biomarkers– and their combinations – of interest for a better understanding of *T. cruzi* infections, but also for a more accurate follow-up of CD in patients. Indeed, identification of parasite strains – and thus their susceptibility to drugs – together with diagnosis of organ damage was possible in a murine model and could be a possible strategy in humans. Our results advocate for the implementation of multi-parametric tools in complex biological systems not only in the field of American trypanosomiasis but beyond, in other complex infectious diseases.

## Methods

### Parasite provenance

All *Trypanosoma cruzi* strains come from the ChagasEpiNet consortium. Epimastigotes were obtained from Michael Miles laboratory (LSHTM, London, UK). For details, see Supplementary Information.

### Epimastigote maintenance and trypomastigote production

Trypomastigote forms obtained by passage on Vero cells of cultured epimastigotes forms were used for production of blood trypomastigotes in IFNγ receptor1-deficient mice (129-*Ifngr1* ^*tm1Agt*^ */J*). For details, see Supplementary Information.

### Mice infection, experimental design

Young adult (6- to 8-wk-old) BALB/c mice were intraperitoneally injected with 2,000 trypomastigotes (200 µl). Mock animals were challenged with 200 µl physiological serum. Parasitemia was monitored for all animals every other day by the Brener method as described elsewhere^37^. For details, see Supplementary Information.

### Ethics statement

This study was carried out in strict accordance with the European Commission legislation for the protection of animals used for scientific purposes (Directives 86/609/EEC and 2010/63/EU). Mice were maintained under pathogen-free conditions at the Centro de Biología Molecular Severo Ochoa (CSIC-UAM) animal facility. The protocol for the treatment of the animals was approved by the “Comité de Ética de Investigación de la Universidad Autónoma de Madrid”, Spain (permits CEI-14-283 and CEI-47-899). Animals had unlimited access to food and water. They were euthanized in a CO2 chamber and all efforts were made to minimize their suffering.

### Organ treatment upon dissection

Upon dissection, blood was taken from the heart and organs were split in different tubes. For DNA and RNA purification, samples were frozen without additional medium in liquid nitrogen. For histology, organs were fixed in 4% paraformaldehyde in 1X PBS. For details, see Supplementary Information.

### Organ weight comparison

For spleen, liver, and heart, organ weight from parasite-challenged animals was normalized to the weight of the whole animal. For details, see Supplementary Information.

### Damage measurement

Damage was determined upon dissection by macroscopic observation and qualitative probing. We categorized these observations into three levels of organ damage: no, low, or high categories (indexes 0, 1, and 2, respectively). For details, see Supplementary Information.

### Parasite load measurement

After proteinase K digestion of tissues, DNA was purified manually by double phenol-chloroform-isoamyl alcohol (25:24:1) extraction, chloroform extraction, and sodium acetate/ethanol precipitation. Quantitative, real-time PCR was performed with references for regression for each strain and tissue. For details, see Supplementary Information.

### Cytokine measurement in serum

Mouse Th1/Th2/Th17/Th22 13plex FlowCytomix Multiplex (eBioscience) was used according to manufacturer’s recommendation. For details, see Supplementary Information.

### Gene expression analysis

After organ homogenisation and acid phenol-chloroform extraction, RNA was retrotranscribed and used for qRT-PCR using 18 S rRNA as normalizer and mock controls as references. For details, see Supplementary Information.

### Statistical analyses

All data were compared using Student’s T-test adjusted to adequate scedasticity assayed by Fisher’s F-test, except for organ damage, compared by Mann-Whitney-Wilcoxon’s non-parametric U test. Correlations were evaluated by Bravais-Pearson’s R linear correlation coefficient.

### Linear discriminant analysis

Damage, parasite load and serum cytokines values, as well as the log_2_ of the fold change for organ weight and gene expression in organs were submitted to linear discriminant analysis (SPSS software, IBM). For details, see Supplementary Information.

### Data availability

The authors will provide any data in their possession upon request.

## Electronic supplementary material

Supplementary Information

Table S1

Table S2

Table S3

Table S4

Table S5

Table S6

Table S7
