# Supplementary material for: A multi-parametric analysis of Trypanosoma cruzi infection: common pathophysiologic patterns beyond extreme heterogeneity of host responses
Source: Sci Rep. 2017 Aug 21;7:8893. doi: 10.1038/s41598-017-08086-8 (PMC5566495; doi:10.1038/s41598-017-08086-8)
Supplement: Supplementary file 1 — Supplementary Information [file 41598_2017_8086_MOESM1_ESM.doc]

# **Supplementary Information**

# Title

A multi-parametric biology analysis of *Trypanosoma cruzi* infection: common pathophysiologic patterns beyond extreme heterogeneity of host responses

# Running title

A multi-parametric analysis of *T. cruzi* infection

# Authors

Julien Santi-Rocca^1,a,*^, Fernando Fernández-Cortés^1,b^, Carlos Chillón-Marinas^1^, María-Luisa González-Rubio^1^, David Martin^2^, Núria Gironès^1,3,c^, Manuel Fresno^1,3,c,*^

# Author affiliation

^1^ Centro de Biología Molecular Severo Ochoa, Consejo Superior de Investigaciones Científicas (CSIC), Universidad Autónoma de Madrid (UAM), Cantoblanco, Madrid 28049, Spain.

^2^ Novancia Business School, 75015, Paris, France

^3^ Instituto Sanitario de Investigación Princesa. Madrid, Spain.

^a^ Current address: Inserm, U1043, Toulouse, F-31300, France

CNRS, U5282, Toulouse, F-31300, France

Université de Toulouse, UPS, Centre de Physiopathologie de Toulouse Purpan (CPTP), Toulouse, F-31300, France

^b^ Current address: Wellcome Trust Centre for Molecular Parasitology, College of Medical, Veterinary and Life Sciences, University of Glasgow, Glasgow, United Kingdom

^c^ Co-senior author

*Corresponding authors

E-mail: [julien.santi-rocca@inserm.fr](mailto:julien.santi-rocca@inserm.fr) (JSR), mfresno@cbm.csic.es (MF)

# Supplementary Figures and Legends


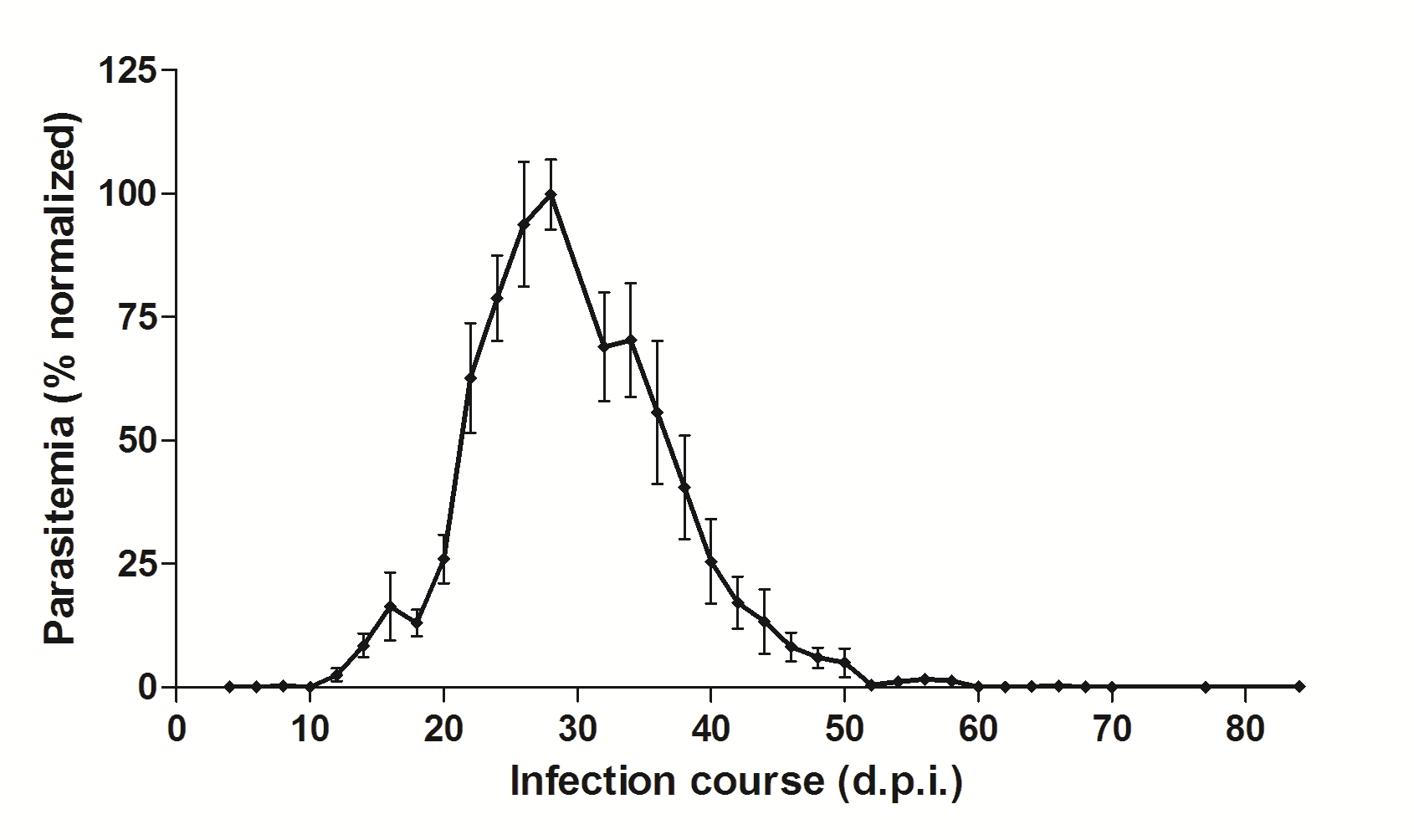


**Figure S1. Parasitemia along course of infection by VFRA parasites.**

Parasites were numbered by Brener method [^37^](#_ENREF_37) every other day in the blood of mice. Results for each animal were normalized to its own maximum parasitemia. Averaged data between animals were then normalized to the maximum for the whole time course. Data are the mean of results from 12 animals until day 28 and 6 until day 84, plotted with the S.E.M.

**
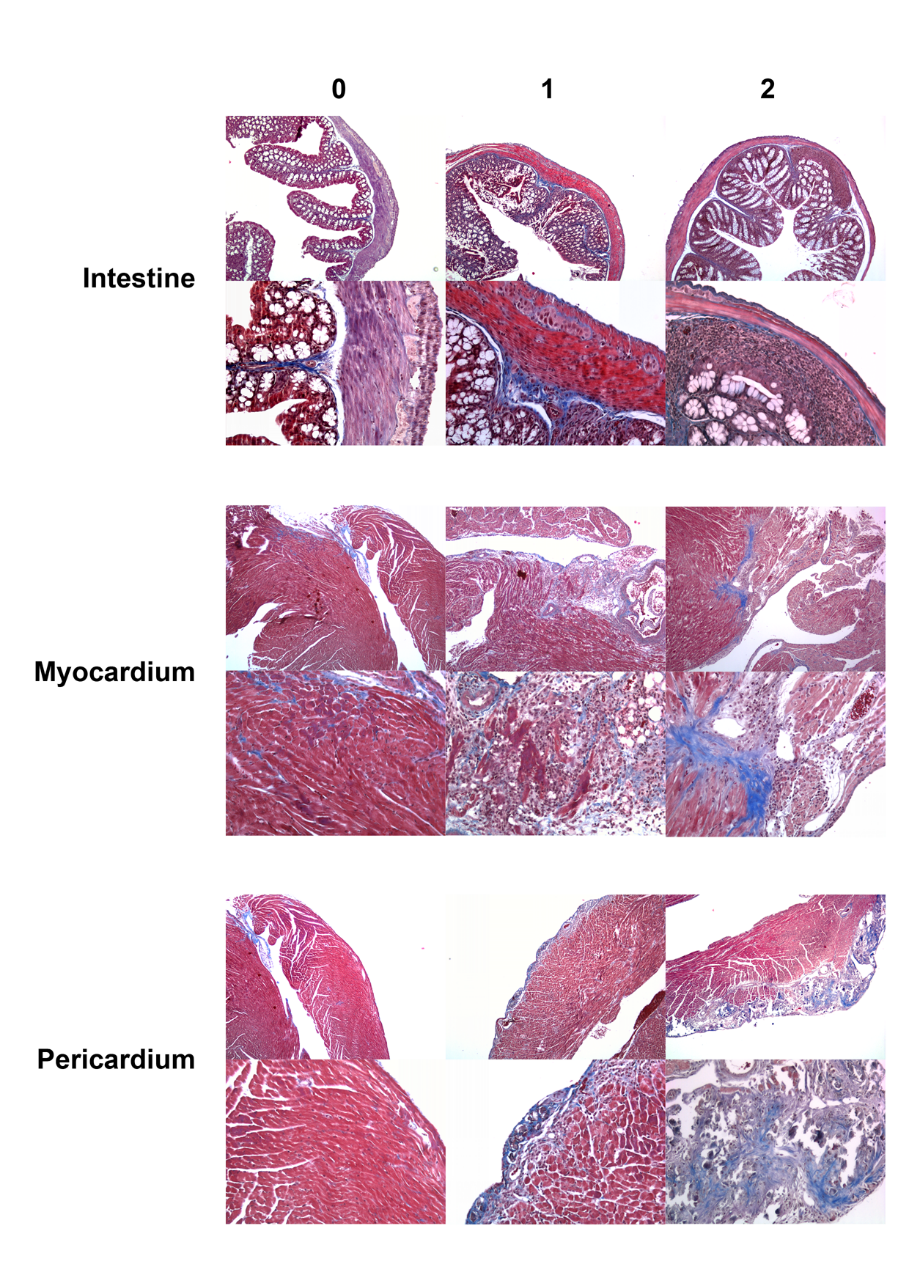
**

**Figure S2. Damage observed macroscopically are linked to histological modifications.**

Histological analysis by Masson’s trichrome staining of organs from control animals (grade 0, left column), or animal infected by VFRA strain with low (grade 1, center column) or high damage (grade 2, right column). For each double row, top micrographs were acquired with a 5X objective and bottom ones with a 20X objective.

In grade 1 colon, a strong staining of fibers is observed at the lamina muscularis mucosae, as well as a mild infiltrate in the mucosa, and a thickening of submucosa and muscularis. In grade 2 colon, the fibrosis is more obvious in both the serosa and the lamina muscularis mucosae, as is the cell infiltrate in the mucosa. Interestingly, the thickness of the submucosa and muscularis is heterogeneous.

Grade 1 myocardium bears a strong cell infiltrate and moderate fibrosis, whereas grade 2 is linked to a strong fibrosis. Similar observations were made for the pericardium.


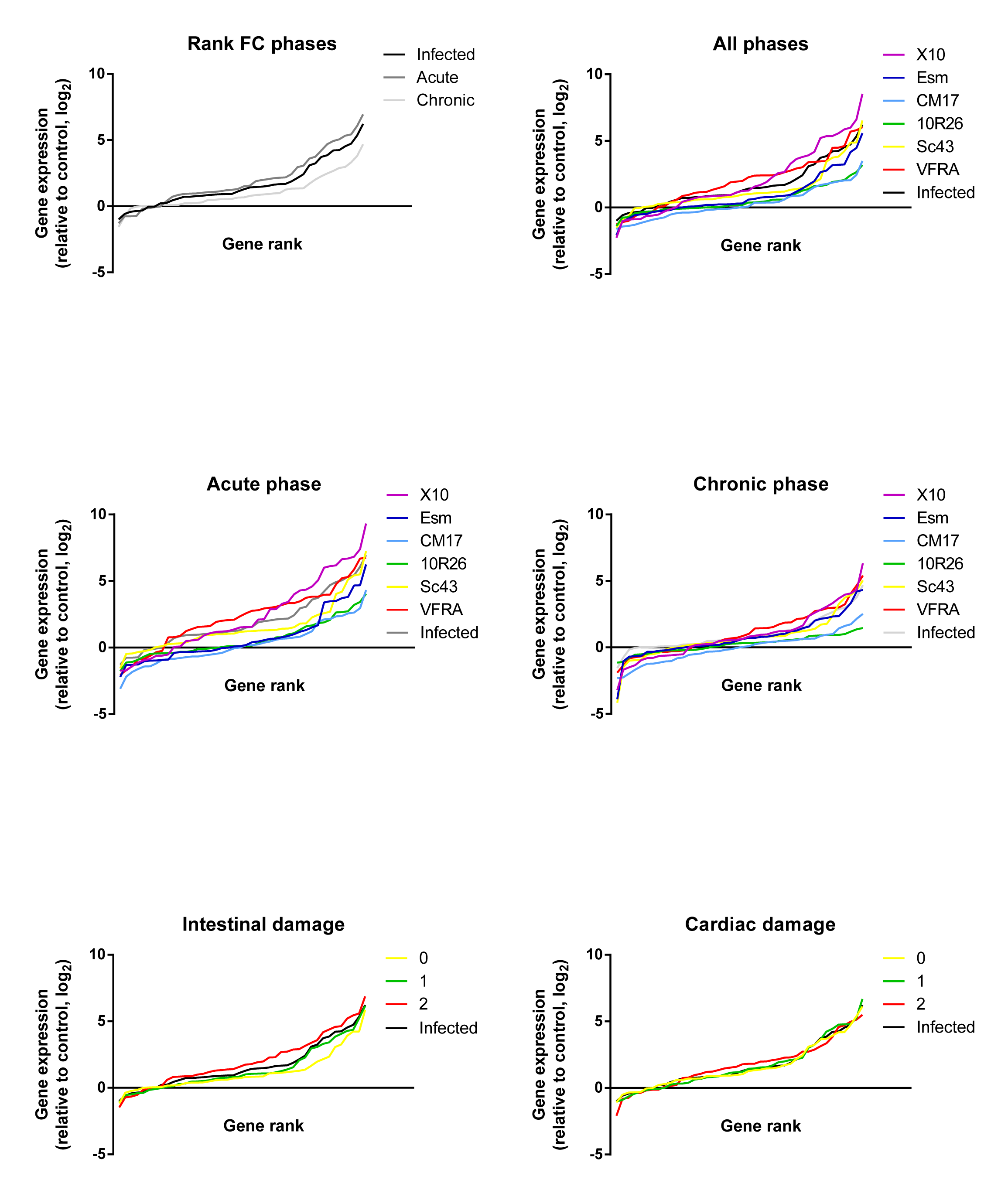


**Figure S3. Patterns of gene expression modulation in various grouping categories.**

Gene expression relative to control (log2) for each gene, ordered on the x-axis by their rank.

**Tables S1 to S6. Detailed data for each measured parameter.**

Top rows group parameters as presented in **Figures 1-4**. Exceptions are the serum cytokines expressed as their absolute concentration (pg/ml) and gene expressions as normalized to controls (whose mean was set to 1) in a linear scale. The third row presents short names for parameters, as used in **Table S7**.

Top table presents data for each animal, arranged in infected and control animals **(Table S1)**, according to phases **(Table S2)**, strains **(Table S3)**, phases and strains **(Table S4)**, intestinal damage grade **(Table S5)**, and cardiac damage grade **(Table S6)**.

Bottom tables show mean values for groups, standard deviations (S.D.), coefficients of variation (C.V.), and p values obtained by corresponding statistical tests. For organ damage, data were compared using Mann-Whitney-Wilcoxon’s non-parametric U test. All other groups, data were compared using Student’s T-test adjusted to adequate scedasticity assayed by Fisher’s F-test. Significance is color-coded: yellow stands for p < 0.05, orange p < 0.01, yellow p < 0.001.

Empty cells stand for unavailable data.

**Table S7. Structure matrix for discriminant analyses.**

The functions calculated by discriminant analysis and used for representation in **Figure 5** are presented together with the proportion of the variance they explain. Data associated to each parameter (short names correspond to the third row in **Tables S1 to S6**) form the canonical structure matrix, revealing the correlation between each variable in the model and the discriminant function. Signs correspond to the contribution towards positive or negative values of the function. For instance, spleen weight (SWe) contributes to negative values in the “Phase” function 1, meaning that splenomegaly discriminates towards groups with negative values, *i.e.* animals in acute phase. Splenic *Arg1* expression (SAr) also has a negative correlation with “Phase” function 1. Downregulation of *Arg1* expression thus correlates with occurrence of chronic phase, which is consistent with the results already described. The intensity of the correlations are color-coded: red colour indicates the strongest contribution to the model (highest absolute values) and green, the lowest correlation (values close to 0).

Only expression of *tnf* in heart did not pass tolerance test, meaning this parameter was, in this case, a linear combination of others; it was excluded from the analysis for strains and cardiac damage discrimination.

# Text S1

To manipulate a reduced set of data, instead of 64 parameters measured in each animal (in addition to damage, strain, and phase), we gave a first attempt with principal component analysis (PCA), which identifies linear combinations of variables likely to make out emerging categories. This approach allowed to reduce dimensions to 12 components, explaining 81.8% of variance in total (data not shown). However, it greatly reduced the discriminant power of further analyses we performed (data not shown). We then decided to treat the whole set of data, conserving all individual parameters.

Discriminant analysis (DA) is a statistical method to find a combination of features that characterizes and separates several classes of objects. It thus allows to express one categorical dependent variable – as strain, phase, or damage – as a combination of continuous independent variables – as the 64 parameters we measured – like in the founding example of Fisher classifying iris species (*Iris setosa*, *I. versicolor*, *I. virginica*; dependent variable) according to the width and length of sepals and petals [^25^](#_ENREF_25).

In our study, linear DA allowed to place each animal strictly in the right group (**Figure 5**), as observed in 1 dimension for phases (**Figure 5B**), 2 dimensions for strains, intestinal and cardiac damages (**Figures 5A, 5D, and 5G**, respectively), although a third dimension permits to explain in total up to 92.7% of the variance of the model, and two additional functions (accounting respectively for 5.4 and 1.9% of the variance, data not shown) allow a flawless classification. **Table S7** shows the correlations between the variables in the model and the discriminant functions; legend for variable names is shown in the first rows of **Table S1**.

### Phase discrimination

Phase discrimination was efficient in one dimension (**Figure 5B**). Function 1 gave negative scores for acute infection and positive values for chronic infections.

Splenic and liver masses were positively correlated with acute phase (Figure 5C), consistent with the stronger hepatosplenomegaly as compared to chronic infection. Overall liver response was also correlated to acute phase, in agreement with the upregulation of most of these genes as compared to chronic phase.

Splenic expression of *Alox15*, *Tgfb1*, *Il12*, *Il4*, and *Nos2* was negatively and weakly correlated to acute phase. Expression of these genes was indeed significantly lower in acute phase relative to chronic phase. However, different patterns emerged: for instance, *Alox15* expression was not modulated in chronic phase while repressed in acute phase, *Nos2* showed no modulation in acute phase and an upregulation in chronic phase, and finally *Il4* was downregulated in both phases, but more strongly in the acute one.

These results, together with detailed statistics presented in **Table S2**, show the interest of DA to interpret the significance and the contribution of each variable to our model.

### Strain discrimination

In an attempt to extract specific markers of each strain, independently of the course of infection, we pooled phases and proceeded to strain discrimination (**Figure 5A**). Discrimination was unexpectedly accurate, with 3 functions explaining 46.9%, 24.7%, and 19.7% of the variance of the model (as presented in **Table S7**). However, 2 functions were sufficient to visually discriminate the 6 strains, as presented in **Figure 5A**.

Function 1 allowed to discriminate CM17 (high positive values) from Esm, 10R26, and Sc43 (low positive values), X10 (low negative values), and VFRA (high negative values). This function is mainly characterized by splenic gene expression (positive values), and cardiac and hepatic gene expression (negative values). An illustrative example is splenic expression of *Il12*, which is downregulated in X10 and VFRA, and consistently positively correlated to Function 1.

The second function permits to gain precision by separating 10R26 (positive values) from Esm and Sc43 (negative values). The structure of this function was characterized by positive values for serum cytokines and splenic gene expression, and negative values for liver and heart gene expression.

### Intestinal damage discrimination

For intestinal damage (**Figure 5D**), the first function (59.8% of variance explained) characterized the grade 2 damage (negative values), opposed to grades 1 and 0 (null values). This function opposed serum and splenic cytokines (positive correlation to Function 1, Figure 5E) to heart, intestine (except *Il6*), and liver gene expression (negative correlation). For instance, intestinal *Arg1* gene expression was negatively correlated to Function 1, as observed by its significantly stronger expression in animals with high intestinal damage as compared to other infected animals.

The second function (40.2% of variance explained) allowed to split animals without damage (negative values) from grades 1 and 2 (null values). Expression of splenic genes and intestinal *Il6*, *Ptgs2*, *Tgfb1*, *Nos2*, and *Il10* genes was correlated to the absence of damage (**Figure 5F**). Interestingly, the latter genes were not statistically modulated in the grade 0 group as compared with grades 1 and 2 together. These data suggest that the local intricate response is difficult to comprehend in a steady analysis, and that the splenic response we evidenced here is protective against parasite-induced intestinal damage.

### Cardiac damage discrimination

For cardiac damage (**Figure 5G**), Function 1 (66.3% of variance explained) efficiently discriminates animals with grade 2 damage (negative values) from others (null values). Expression of most splenic genes was positively correlated to this function (**Figure 5H**). Most serum cytokines were negatively correlated to Function 1, in particular IL-4, the only cytokine to be significantly more concentrated in the sera of animals with high cardiac damage as compared to other infected animals.

Function 2 (33.7% of variance explained) allowed to split animals with low cardiac damage (negative values) from other infected animals (null values). Most liver genes and serum cytokines were positively correlated to this function (**Figure 5I**). However, some factors were strongly, negatively correlated to Function 2, thus linked to grade 1 cardiac damage. Two of these parameters were significantly upregulated in animals with low cardiac damage: circulating TNF concentration and heart *Alox15* expression. Serum TNF concentration was indeed higher in grade 1 cardiac damage as compared to other infected animals. However, its source or dynamics in serum still need further investigation to be elucidated.

ALOX15 (arachidonate 15-lipoxygenase) is an enzyme generating a spectrum of bioactive lipid mediators. The substrates of this enzyme in the cardiac context during infection is still unknown; however, the differential modulation of its gene suggests a modification in cell communication. *Alox15* was the only significantly modulated gene in heart of animals with low cardiac damage as compared to other infected animals. Surprisingly, all the genes measured in the heart were negatively correlated to Function 2. This highlights that heart response is linked to damage and that different types of immune polarizations may be involved, as proposed in complementary models of inflammation- or autoimmunity-driven damage. Responses may be exclusive from each other but may also be spatially or temporally compartmentalized in the same animal, thus explaining the complexity and the variability of the parameters that were measured. This intricate analysis underlines the limits of classical univariate and central statistical approaches.

# Supplementary Experimental Procedures

## Parasite provenance

All *Trypanosoma cruzi* strains come from the ChagasEpiNet consortium. Epimastigotes were obtained from Mickael Miles laboratory (LSHTM, London, UK).

Silvio X10/1 (X10) parasites (TcI) were obtained from a patient in Belém, Brazil. Esmeraldo (Esm) parasites (TcII) were obtained from a patient in São Felipe, Brazil. CM17 parasites (TcIII) were purified from *Dasypus sp.* in Meta, Colombia. 10R26 parasites (TcIV) were purified from *Aotus sp.* in Santa Cruz, Bolivia. Sc43 parasites (TcV) were purified from *Triatoma infestans* in Santa Cruz, Bolivia. VFRA1 (VFRA) parasites (TcVI) were purified from *Triatoma infestans* in Francia, Chile.

## Epimastigote maintenance and trypomastigote production

Epimastigote forms were maintained in logarithmic growth by continuous passage in liver infusion tryptose (LIT) medium supplemented with 10% serum and 10 mg/L hemin in closed flasks at 28°C. These parasites were used for passage on Vero cells, cultured in Dulbecco’s modified Minimal Essential Medium (DMEM) supplemented with 10% v/v heat-inactivated foetal bovine serum (FBS). Infection was achieved by a 24-hour incubation in DMEM supplemented with 20% FBS. Parasites that did not infect cells were washed away with medium supplemented with 20% FBS. Cultures were then maintained in DMEM with 10% FBS until trypomastigotes could be retrieved in the medium.

Before infection of immunocompetent mice, blood trypomastigotes were produced in IFNγ receptor1-deficient mice (129-*Ifngr1^tm1Agt^/J*, from The Jackson Laboratory). Blood was taken from CO_2_-asphixied animals and immediately diluted in cold physiological serum. After a centrifuge at 2,000xg for 5 minutes to remove serum, pellet was resuspended in physiological serum and centrifuged at 200xg for 5 minutes. Above the lower phase containing erythrocytes, the interphase contained leukocytes and parasites. After a 5-minute incubation at room temperature, parasites in the upper phase were counted thrice in a Neubauer hemocytometer and diluted in physiological serum at a concentration of 10^4^ parasites/ml.

## Mice infection, experimental design and ethics statement

Young adult (6- to 8-wk-old) BALB/c mice were purchased from Charles River Laboratories. Blood trypomastigotes were intraperitoneally injected at a dose of 2,000 (200 µl). Mock animals were challenged with 200 µl physiological serum. For each parasite strain, 4 littermates were challenged with parasites and 4 other mice were mock challenged (also denominated “uninfected” or “control” animals). Parasitemia was monitored for all animals every other day by the Brener method as described elsewhere [^37^](#_ENREF_37)

. After 28 days of infection, corresponding to acute phase, 2 infected and 2 mock, randomly chosen mice were sacrificed. The 4 remaining mice were sacrificed at 84 days, corresponding to the chronic phase for animal challenged with *T. cruzi*.

Three different groups of infection were done for each strain, leading to 6 animals in acute phase and 6 in chronic phase, with corresponding controls. For reduction purposes, some mock controls were shared among strains, hence their reduced number in **Table S1**.

This study was carried out in strict accordance with the recommendations of Spanish legislation and the European Commission legislation for the protection of animals used for scientific purposes (Directive 2010/63/EU). All mice were maintained under pathogen-free conditions in the animal facility at the Centro de Biología Molecular, Universidad Autónoma de Madrid (Madrid, Spain). The animal protocol was approved by the Ethical committee of the Universidad Autónoma de Madrid. Animals had free access to food and water and were handled in compliance with European norms. Mice were euthanized in a CO_2_ chamber, and all efforts were made to minimize suffering.

## Organ treatment upon dissection

After weighting the animal, blood was taken from the heart. After coagulation, serum was retrieved by centrifugation.

Organs were perfused with ice-cold 1X PBS and immediately treated: they were weighted (spleen, liver, heart), homogenised with scissors on ice. Organs were split in different tubes for the subsequent treatment and accurately weighted with a precision scale before freezing in liquid nitrogen.

For histology, organs were fixed in 4% paraformaldehyde in 1X PBS for 24 hours at 4°C before paraffin inclusion and Masson’s trichrome staining at the histology service of the Centro Nacional de Biotecnología (Madrid).

## Organ weight comparison

For spleen, liver, and heart, organ weight from parasite-challenged animals was normalized to the weight of the whole animal. Then, this result was reported to the mean value of the weight of paired mock animals, leading to the mass index presented in the study.

## Damage measurement

Damage was determined upon dissection by macroscopic observation and qualitative probing. For the heart, white stripes on the cardiac muscle and remarkable changes in texture and appearance were considered markers of damage. For the intestine, loss of elasticity of the colon leading to rupture upon extension, changes in the volume and tissue colour were indicators used for damage indexing. We categorized these observations into three levels of organ damage: no, low, or high categories (indexes 0, 1, and 2, respectively).

## Parasite load measurement

Organs were frozen and thawed in 3 ml lysis buffer (100 mM NaCl, 10 mM Tris pH 8.0, 1% SDS, 1 mM CaCl_2_) *per* mg of tissue. Lysis was achieved by incubation at 55°C with 500 µg proteinase K *per* g of tissue, until total dissolution. Final volume was adjusted to 200 mg tissue / ml. RNA was digested by diluting 50 µl (equivalent to 10 mg tissue) with 450 µl Tris 10 mM pH 8.0, supplemented with 50 µg RNase A and incubated at 37°C for 1 h. DNA was purified by double phenol-chloroform-isoamyl alcohol (25:24:1) extraction, chloroform extraction, and sodium acetate/ethanol precipitation. Samples were then resuspended in water and adjusted to 50, 5, and 0.5 ng DNA / µl.

References for regression were done for each strain and each tissue. Epimastigote forms of parasites were washed in 1X PBS and resuspended in lysis buffer with 50 µg proteinase K *per* ml in 10-fold dilutions ranging from 2x10^3^ to 2x10^7^ parasites / ml. After a 1-hour incubation, 50 µl tissue from mock-challenged animals was spiked with 50 µl parasite lysate (or lysis buffer), added to 400 µl 10 mM Tris pH 8.0 with 50 µg RNAse A. Subsequent steps were identical to those previously described. Standards were resuspended in water and adjusted to 100, 10, 1, and 0.1 ng DNA / µl.

For qPCR (ABI PRISM 7900HT, Applied Biosystems), 1 µl resuspension was used for 25 µl final volume, and experimental procedure was described elsewhere, in triplicates (*22*).

Data were treated using a bidimensional regression. First, mammalian 18s rDNA was used to identify the 2 closest total DNA dilutions of unspiked standards surrounding the value obtained for each dilution of the infected tissue. Then, for each total DNA dilution, Δ*C_T_* = f(log_10_ (parasite concentration)) was plotted, where Δ*C_T_* = *C_T_*_(parasite DNA)_ - *C_T_*_(18srDNA)_. This function was used for the linear regression using the values of Δ*C_T_* obtained for infected tissues, obtaining 2 parasite concentrations (with the two standard values) for each of the 3 dilutions of the infected samples. These 6 values were averaged and used as the unique final result for the animal.

## Cytokine measurement in serum

Mouse Th1/Th2/Th17/Th22 13plex FlowCytomix Multiplex (eBioscience) was used according to manufacturer’s recommendation. Bead fluorescence was read on a FACSCanto II cytometer (BD) and data were treated with FACSdiva (BD) and subsequently with FlowCytomix software (eBioscience). Data are presented in **Tables S1 to S6** as the concentration (pg/ml) in the serum and in **Figure 3** as the ratio to highest group mean for representation purposes.

## Gene expression analysis

After organ homogenisation by Polytron (Kinematica) in TRIzol reagent (Invitrogen), RNA was purified using acid phenol-chloroform extraction. Retrotranscription was performed with High Capacity cDNA Archive Kit (Applied Biosystems) according to manufacturer’s recommendations. Gene amplification was performed in TaqMan Universal PCR Master Mix (Applied Biosystems) using TaqMan MGB probes for the indicated genes on ABI PRISM 7900 HT instrument (Applied Biosystems). Gene expression was calculated by the -ΔΔC*_T_* method, using 18S rRNA as normalizer and mock controls as references. Results are presented as the fold change relative to controls (**Tables S1 to S6**) or the log_10_ of this value (**Figure 4**).

## Linear discriminant analysis

All data were adjusted to produce a set in which the values of the control group was 0, with negative values for lower indices than the controls, and positive values for the opposite case. This corresponded to unchanged data from **Tables S1 to S6** for damage, parasite load and serum cytokines. Organ weight and gene expression in organs were expressed as the log_2_ of the fold change mentioned in **Tables S1 to S6**. For further analysis, missing data from a given parameter were replaced by regression (“Automatic Linear Modeling” in SPSS software, IBM) based on the most positively and negatively correlated parameters (upper and lower deciles, Pearson’s R) in the specific phase and strain group. Then, the whole substituted set was submitted to linear discriminant analysis (SPSS software, IBM). The discriminant procedure for each of the “grouping variable(s)” (strain, phase, damages) was performed entering simultaneously the 64 parameters as “independent variables”.

A tolerance test for multicollinearity was run to check the relevance of including each of these independent variables. The coefficients for each independent variable of the structure matrix presented in **Table S7** can be interpreted like in multiple regression, *i.e.* as an index of the importance of each predictor. The sign indicates the direction of the relationship; the absolute value, the strengh of predictive power of the parameter.
